# Can I Discharge My Stroke Patient Home After Inpatient Neurorehabilitation? LIMOS Cut-Off Scores for Stroke Patients “*Living Alone” and “Living With Family”*

**DOI:** 10.3389/fneur.2020.601725

**Published:** 2020-11-25

**Authors:** Beatrice Ottiger, Dirk Lehnick, Tobias Pflugshaupt, Tim Vanbellingen, Thomas Nyffeler

**Affiliations:** ^1^Neurocenter, Luzerner Kantonsspital, Lucerne, Switzerland; ^2^Clinical Trial Unit Central Switzerland, University of Lucerne, Lucerne, Switzerland; ^3^ARTORG Center for Biomedical Engineering Research, Gerontechnology and Rehabilitation Group, University Bern, Bern, Switzerland

**Keywords:** stroke, living alone, living with family, discharge destination, ADL, LIMOS

## Abstract

**Background:** Discharge planning of stroke patients during inpatient neurorehabilitation is often difficult since it depends both on the patient's ability to perform activities of daily living (ADL) and the social context. The aim of this study was to define ADL cut-off scores using the Lucerne ICF-based multidisciplinary observation scale (LIMOS) that allow the clinicians to decide whether stroke patients who “live alone” and “live with a family” can be discharged home or must enter a nursing home. Additionally, we investigated whether age and gender factors influence these cut-off scores.

**Methods:** A single-center retrospective cohort study was conducted to establish cut-off discharge scores for the LIMOS. Receiver-operating-characteristics curves were calculated for both patient groups “living alone” and “living with family” to illustrate the prognostic potential of the LIMOS total score with respect to their discharge goals (home alone or nursing home; home with family or nursing home). A logistic regression model was used to determine the (age- and gender-adjusted) odds ratios of being released home if the LIMOS total score was above the cut-off. A single-center prospective cohort study was then conducted to verify the adequacy of the cut-off values for the LIMOS total score.

**Results:** A total of 687 stroke inpatients were included in both studies. For the group “living alone” a LIMOS total score above 158 indicated good diagnostic accuracy in predicting discharge home (sensitivity 93.6%; specificity 95.4%). A LIMOS total cut-off score above 130 points was found for the group “living with family” (sensitivity 92.0%; specificity 88.6%). The LIMOS total score odds ratios, adjusted for age and gender, were 292.5 [95% CI: (52.0–1645.5)] for the group “living alone” and were 89.4 [95% CI: (32.3–247.7)] for the group “living with family.”

**Conclusion:** Stroke survivors living alone needed a higher ADL level to return home than those living with a family. A LIMOS total score above 158 points allows a clinician to discharge a patient that lives alone, whereas a lower LIMOS score above 130 points can be sufficient in a patient that lives with a family. Neither age nor gender played a significant role.

## Introduction

The planning of discharge during inpatient neurorehabilitation in stroke patients is a dynamic process and critically depends on the patients' functional progress and ability to perform activities of daily living (ADLs). In addition to performance in ADL, various factors such as demographic background, age, gender, access to municipal organizations and the social context also plays an important role in deciding whether a patient can return home or must enter a nursing home (1, 2). Previous studies emphasized that one of the strongest factors of being discharged home or not is the living situation [i.e., if a patient lives alone or with a family (3–5)]. Stroke survivors often require the assistance of family caregivers to cope with their physical, cognitive and emotional deficits at home (6, 7). After inpatient neurorehabilitation, patients who have a caregiver at home are therefore more likely to be discharged home (3, 4) than patients living alone (1, 3, 4, 8). For instance, although stroke survivors living alone can partially be supported by community or professional organizations, they lack the twenty-four-seven support of a person living in the same household. This suggests that to be discharged home, a stroke patient living alone must show better performance in the activities of daily living (ADLs; e.g., moving around at home, preparing a meal etc.) than a stroke patient living with a family. This is particularly relevant for Switzerland, since a third of the Swiss population lives alone (9). This trend is also steadily increasing worldwide (10, 11).

Therefore, it is important to continuously assess ADL performance of inpatients during neurorehabilitation and to estimate performance levels sufficient for returning home. To accurately measure the ability of ADL performance according to the International Classification of Functioning, Disability and Health (ICF) framework set by the World Health Organization (WHO), we recently developed the Lucerne ICF-based Multidisciplinary Observation Scale (LIMOS) and validated it in stroke patients (12). Using this scale, patients with stroke are observed with respect to their activity ability by health professionals involved in their neurorehabilitation (nurses, physiotherapists, speech therapists, occupational therapists, as well as neurologists). This will be done in the first 72 h after admission, then weekly during the stay and in the last 72 h before discharge from inpatient neurorehabilitation. The observations are structured and consist of 45 basic and instrumental ADL items based on the (ICF) framework, which are categorized in four factors (interpersonal activities, motor and self-care; communication; knowledge and general tasks; and domestic life). The LIMOS measures the level of assistance needed from the health professionals, with higher scores representing more independence (12). As each discipline rates their own subpart within the whole LIMOS, it is easy and short to conduct and requires only 5 to 10 min per discipline. The advantage of LIMOS is that it is more comprehensive and more sensitive than the Functional Independence Measure (FIM) and Barthel Index (BI) (13). In addition, the LIMOS scale shows neither floor nor ceiling effects at admission and discharge, in contrast, to the FIM and BI (12, 13). Using the LIMOS thus allows the patients' activity levels to be assessed comprehensively.

Based on previous studies suggesting that ADL performance and living situations are crucial factors to be able to return home after stroke neurorehabilitation (1, 5), the aim of the present study was to define LIMOS cut-off scores in ADL performance for stroke patients living alone and those living with a family. Such scores would provide clinicians a tool that facilitates the decision concerning the discharge destination during inpatient rehabilitation. A second aim was to verify whether the factors age and gender influence these cut-off scores because previous studies have found that older people and women had a worse prognosis for returning home after stroke (1, 14, 15).

## Methods

### Study Design

1. A single-center retrospective cohort study to establish cut-off values for the LIMOS total score
2. A single-center prospective cohort study to verify the cut-off values for the LIMOS total score.

### Sample

A sample of 555 inpatients with stroke diagnosis recruited between January 2014 and February 2019 was included in the first study. A separate second sample of 132 inpatients, admitted between April 2018 and May 2020 was prospectively included in order to verify the findings of the first study. All patients were admitted to the neurorehabilitation at the Neurocenter Luzerner Kantonsspital (LUKS) in Switzerland.

Inclusion criteria: Patients who had a first-ever cerebral stroke of any type confirmed by brain computed tomography (CT) or magnetic resonance imaging (MRI) were eligible for the study.

Exclusion criteria: Patients who had additional psychiatric diseases or who were admitted for re-rehabilitation were excluded. Patients who lived in a nursing home or had assistance by a caregiver or external organization prior to admission were excluded.

### Data Collection

#### Socio-Demographic Data

Medical and demographic data such as age, gender, diagnosis, length of stay, time since stroke, in neurorehabilitation was collected from patients' charts.

#### Living Situation Category

The living situation prior admission was evaluated with a structured interview. Within the 1st week of neurorehabilitation, a nurse asked patients about their living situation before stroke (i.e., the so-called living situation category prior admission). If patients were not able to attend the interview, their relatives were asked. The group “living with family” included patients with a spouse or a partner or other family member in the same household, in contrast to the group “living alone.” Patients who lived in a nursing home or had assistance by a caregiver or external organization prior to admission were excluded. Before the end of the neurorehabilitation stay, a meeting was held with the neurologists, the responsible nurses, and therapists as well as with the patient and his relatives to determine the place of discharge. The patient LIMOS total score was used a basis of the discussion. The living situation after discharge was then assigned by the interdisciplinary team and the patient to one of the following categories: “home alone” or “nursing home” for the group “living alone” and “home with family” or “nursing home” for the group “living with family.”

### Outcome

#### Lucerne ICF-Based Multidisciplinary Observation Scale (LIMOS)

Each stroke patient was assessed with LIMOS by a multidisciplinary team (nurses, occupational therapists, physiotherapists, speech therapists, neurologists) within the first 72 h after admission and in the last 72 h before discharge from inpatient neurorehabilitation. The LIMOS consist of 45 basic and instrumental ADL's, selected from the ICF chapters. LIMOS measures the level of assistance needed from the health professionals during activities of daily life (ADL), with higher scores representing more independence (12, 13). The total score ranges from 45 to 225. Every item of LIMOS is rated as follows: 1 = patient is not able to fulfill a task or needs assistance up to 75% (i.e., complete assistance); 2 = patient is able to fulfill tasks with assistance of 25–75% (i.e., severe assistance); 3 = patient is able to fulfill tasks with assistance <25% or under supervision (i.e., moderate assistance); 4 = patient is able to fulfill tasks independently but needs more time and/or with auxiliary materials, aids (i.e., slight assistance); 5 = patient is able to fulfill tasks independently (i.e., no assistance needed). The 45 items are separated into four subscales (factors): LIMOS interpersonal activity, motor and self-care, LIMOS communication, LIMOS knowledge and general tasks and LIMOS domestic life (12). The items to be assessed are assigned to individual professional groups (nursing care: LIMOS items 1, 13–21, 40; physiotherapy: LIMOS items 2–12; occupational therapy: 26–39, 41–45; speech therapy or neurologists: LIMOS items 22–25). The 45 LIMOS items are presented in the Supplementary File 2.

The LIMOS has been validated in two former studies (12, 13). A high internal consistency (0.98) was found for the LIMOS. The inter-rater reliability analysis showed moderate to almost perfect agreement in most domains (Kappa 0.41–0.92) (12). Significant correlations between LIMOS and FIM indicate that both measure the same construct but the LIMOS is more comprehensive (12). The LIMOS show neither floor nor ceiling effects at admission and discharge (all <15%) (13). The LIMOS motor and LIMOS cognition and communication subscales were more responsive, expressed by higher effect sizes (ES) (ES = 0.65, SRM = 1.17, and ES = 0.52, standardized response means (SRM) = 1.17, respectively) as compared with FIM motor (ES = 0.54, SRM = 0.96) and FIM cognition (ES = 0.41, SRM = 0.88) and the BI (ES = 0.41, SRM = 0.65). Changes in the LIMOS values of entry and exit from neurorehabilitation correlate significantly (p < 0.0001) with changes in the FIM motor and FIM cognition scales, indicating good responsiveness (13).

### Statistical Analysis

Descriptive statistics for qualitative data were expressed as number of cases and percentages.

Descriptive statistics for quantitative data included median, first and third quartile (Q1, Q3) and range.

Demographics and clinical characteristics were compared within the two groups “living alone” and “living with family” regarding their discharge destinations (home alone or nursing home; home with family or nursing home, respectively) using Wilcoxon rank-sum test for continuous variables (age, LIMOS). Fisher's exact tests were applied for associations with nominal variables (discharge destination, gender).

Receiver operating characteristics (ROC) curves were computed for the group “living alone” and the group “living with family” in order to illustrate the prognostic potential of the LIMOS total score at discharge regarding their discharge destinations (home alone or nursing home; home with family or nursing home, respectively). Cut-off values were calculated, utilizing the method by Liu (16) and confirmed by the method of Youden (17) and as well as by finding the cutpoint on the ROC curve closest to (0, 1). Furthermore, sensitivity, specificity, negative and positive predictive values and the area under the curve (AUC) were calculated in order to identify the best cut-off scores regarding the discharge destination (home alone or nursing home; home with family or nursing home). A logistic regression model was used to determine the odds ratios (adjusted for age and gender) for the chance of being released home if the LIMOS total score is above the cut-off. Since the LIMOS total score is considered to be a validated ICF-based metric comprehensively assessing the patient's activity levels, no further factors rather than age and gender were added to the initial model. It is assumed that these factors (LIMOS, age, gender) are representing a supportive input, feeding and driving the situation and the conversations between patients, families and treating neurologists when it comes to the decision on the discharge destination. Utilizing Akaike's information criterion, it was further investigated whether a reduced model can predict the discharge destination (e.g., whether age and gender—in addition to the impact provided by a LIMOS total score at discharge above or below the cut-off—still contributed a meaningful independent explanatory value to the discharge destination decision).

As a validation, the adequacy of the cut-off values was verified in a separate second sample (study 2). Applying the cut-off values for the LIMOS total score at discharge, as determined based upon the first sample, diagnostic metrics (sensitivity, specificity, positive and negative prognostic value) were calculated for the group “living alone” and the group “living with family,” using the data of the second sample.

In addition, we estimated probabilities to reach discharge destination “home” by group (“living alone” or “living with family”), derived from a logit model explaining discharge destination by the LIMOS total score at discharge.

Statistical analyses were performed using STATA (Version 16.1 or later, StataCorp, College Station, Texas, USA).

## Results

### Study 1

#### Characteristics of the Patient Sample

555 patients with subacute stroke were included in this study (41.6% females) (Table 1). At admission, median time after stroke onset was 9 days (Q1: 7, Q3: 15, range 2–95 days). Three hundred and ninety-five (71.2%) patients suffered ischemic stroke, 160 (28.8%) hemorrhagic stroke.

**Table 1:** Demographic and clinical characteristics of the patients overall and by group (living alone vs. living with family).

**Variable**	**Overall (n = 555)**	**Group living alone (n = 137)**	**Group living with family (n = 418)**	***p*-value**
	**Median (Q1, Q3) range**	**Median (Q1, Q3) range**	**Median (Q1, Q3) range**	
Discharged home	468/555 (84.3%)	94/137 (68.6%)	374/418 (89.5%)	*p* < 0.001^a^
Gender	231 females (41.6%)	71 females (51.8%)	160 females (38.3%)	*p* = 0.007^a^
	324 males (58.4%)	66 males (48.2%)	258 males (61.7%)	
Age, years	68 (57, 77) 18–94	72 (60, 80) 24–94	67 (57, 76) 18–90	*p* = 0.002^b^
LIMOS total at admission	136.1 (95.2, 169.5) 45.0–221.3	132.9 (99.6, 174.6) 45.0–221.0	136.8 (93.6, 168.1) 45.0–221.3	*p* = 0.87^b^
LIMOS total at discharge	179.7 (150.5, 198.5) 45.0–225.0	180.8 (137.3, 198.3) 45.0–225.0	179.1 (152.4, 198.6) 45.3–224.3	*p* = 0.68^b^

a *Fisher's exact test*.

b *Wilcoxon rank-sum test*.

#### Overall Characteristics of the Two Groups “Living Alone” and “Living With Family”

**Figure 1:**
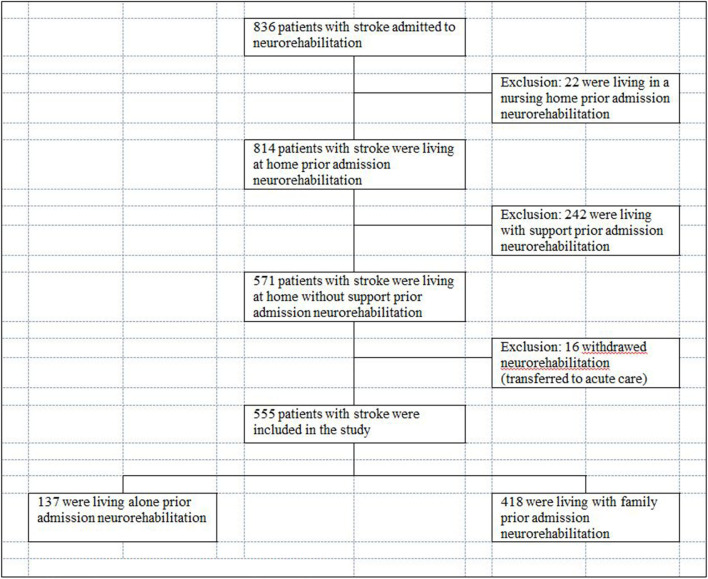
Flow chart—patient sample study 1.

**Figure 2:**
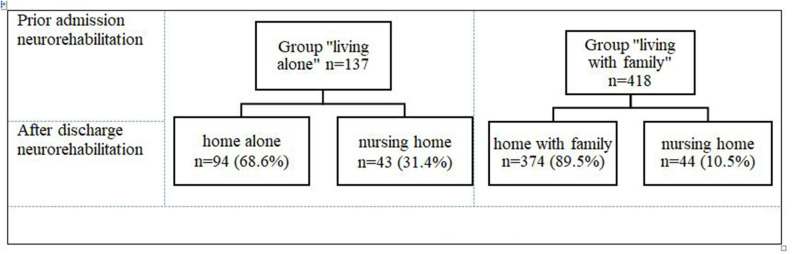
Living situation prior admission and after discharge in-patient neurorehabilitation.

Prior to the admission for an inpatient neurorehabilitation, 137 (24.7%) of patients were in the group “living alone” and 418 (75.3%) were in the group “living with family” (Figure 1). At admission and at discharge, both groups were comparable regarding their ADL impairments (Table 1). The patients in the group “living alone” were significantly older than those in the group “living with family” (Table 1). Additionally, there was a significant group difference in gender frequencies: women were overrepresented in the group “living alone,” men in the group “living with family” (Table 1). Approximately three times more patients from the group “living alone” were discharged to nursing homes (31.4%; *n* = 43/137) than from the group “living with family” 10.5% (*n* = 44/418) (Figure 2).

Given the expected differences between the groups “living alone” and “living with family” with regard to the handling of decisions on whether a patient can be discharged home, any further considerations, including the determination of those cut-offs for the LIMOS total scores at discharge that have supported the discharge destination decisions, will be performed within both groups separately.

#### In Both Groups “Living Alone” and “Living With Family,” Younger Patients and Those Who Had Higher ADL Performances Scores Were Discharged Home

The patients who were discharged home were significantly younger and had higher LIMOS ADL scores than those who went to a nursing home (Table 2). The gender distribution in both groups showed no differences between the discharge destinations (Table 2).

**Table 2:** Demographics and clinical characteristics of the patients by discharge destination (per group).

**Variable**	**Discharge destination “home” (*n* = 94)**	**Discharge destination “nursing home” (*n* = 43)**	***p*-value**
	**Median (Q1, Q3) range**	**Median (Q1, Q3) range**	
**Group “living alone” (*****n*** **=** **137)**			
Gender	47 females (50.0%)	24 females (55.8%)	*p* = 0.58^a^
	47 males (50.0%)	19 males (44.2%)	
Age, years	68.5 (54, 79) 24–90	76 (70, 84) 43–94	*p* < 0.001^b^
LIMOS total at admission	157.3 (131.2, 180.8) 50.0–221.0	81.5 (55.6, 110.0) 45.0–180.3	*p* < 0.001^b^
LIMOS total at discharge	193.3 (178.6, 204.4) 101.7–225.0	121.8 (77.8, 142.0) 45.0–189.8	*p* < 0.001^b^
**Variable**	**Discharge destination “home” (*****n*** **=** **374)**	**Discharge destination “nursing home” (*****n*** **=** **44)**	***p*****-value**
	**Median (Q1, Q3) range**	**Median (Q1, Q3) range**	
**Group “living with family” (*****n*** **=** **418)**			
Gender	142 females (38.0%)	18 females (40.9%)	*p* = 0.74^a^
	232 males (52.0%)	26 males (59.1%)	
Age, years	67 (57, 75) 18–89	70.5(61.5, 80.5) 31–90	*p* = 0.023^b^
LIMOS total at admission	143.4 (109.4, 171.3) 45.0–221.3	60.6 (50.3, 80.5) 45.0–126.3	*p* < 0.001^b^
LIMOS total at discharge	184.0 (164.0, 200.4) 66.3–224.3	91.8 (71.8, 115.2) 45.3–164.5	*p* < 0.001^b^

a *Fisher's exact test*.

b *Wilcoxon rank-sum test*.

#### The Group “Living Alone” Had a Higher LIMOS Total Discharge Cutpoint Than the Group “Living With Family”

**Figure 3:**
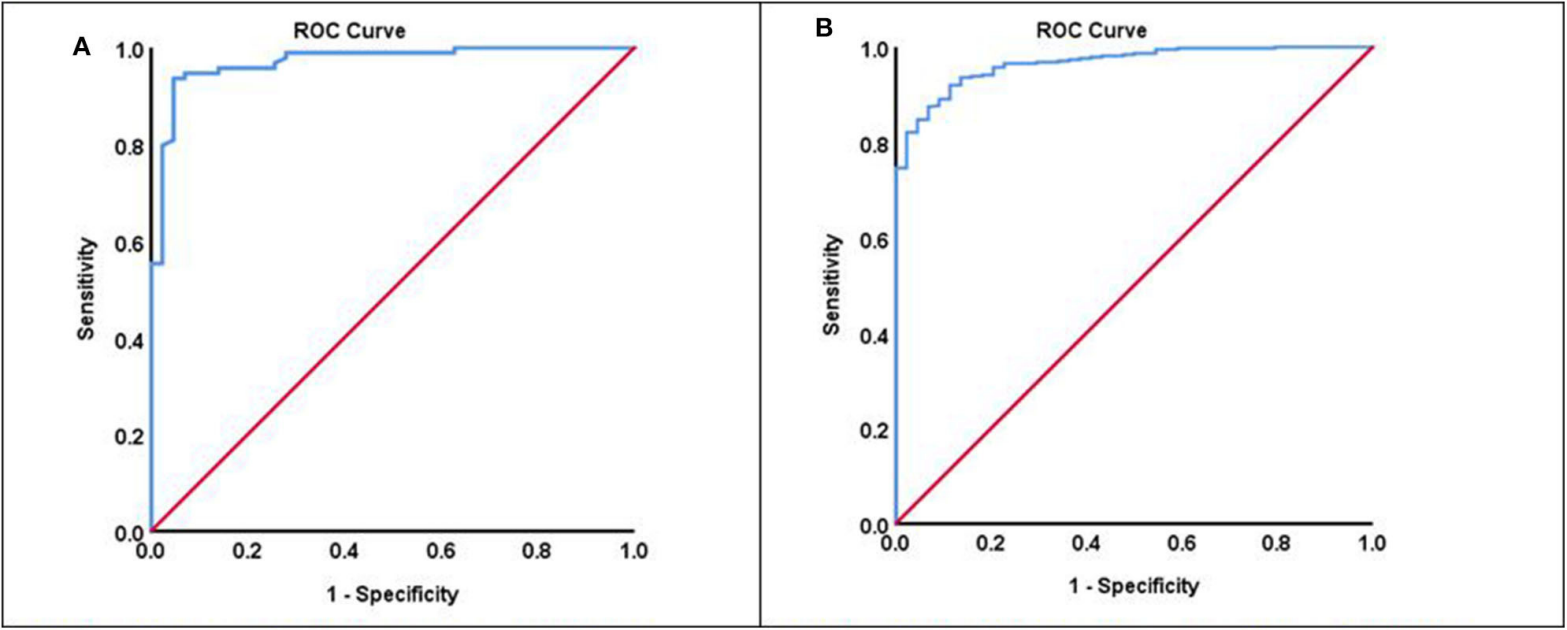
**(A)** ROC curve of the group “living alone” (ROC AUC: 0.971). **(B)** ROC curve of the group “living with family” (ROC AUC: 0.966).

We calculated receiver operation characteristic (ROC) curves for both groups to illustrate the prognostic potential of the LIMOS total score for the discharge destinations home or nursing home (Figures 3A,B), providing excellent ROC AUC of 0.971 for the group “living alone” and 0.966 for the group “living with family.”

For the group “living alone,” a LIMOS total score below 158 was found to be predictive for discharged to a nursing home; LIMOS total scores above that cut-off score indicated good diagnostic accuracy in predicting discharge home. Based upon a cut-off value of 158, a high sensitivity (93.6%) and specificity (95.4%) can be obtained (Table 3).

**Table 3:** Best predictive LIMOS total discharge cut off scores (overall) for discharge destination home.

**Discharge destination “home”**	**Cut-off**	**Sensitivity**	**Specificity**	**PPV**	**NPV**
		**(95% CI)**	**(95% CI)**	**(95% CI)**	**(95% CI)**
**Overall (*****n*** **=** **555)**					
Group “living alone” (*n* = 137)	>158	0.936 (0.866–0.976)	0.954 (0.842–0.994)	0.978 (0.922–0.997)	0.872 (0.743–0.952)
Group “living with family” (*n* = 418)	>130	0.920 (0.888–0.945)	0.886 (0.754–0.962)	0.986(0.967–0.995)	0.565 (0.440–0.684)

In contrast, a remarkably lower LIMOS total cut-off score was found for the group “living with family:” Scores above 130 points indicated diagnostic accuracy in predicting discharge home, while a LIMOS total score below 130 was found to be predictive for discharged to a nursing home. This cut-off value was more sensitive (92.0%) than specific (88.6%) (Table 3).

For both groups, the chosen cut-points resulted in very high positive predictive values (97.8 and 98.6%, respectively). I.e., patients achieving a LIMOS score above the cut-off were discharged home with a fairly high degree of certainty. However, the somewhat lower negative predictive value (56.5%) in the group “living with family” means that a still quite high proportion of patients were ultimately discharged home even if the LIMOS score was below the cut-off.

A logistic regression of the binary outcome (discharge destination “home” vs. “nursing home”) resulted in very high odds ratios for the chance of being released home if the LIMOS total score is above the cut-off (Table 4). The corresponding odds ratios, adjusted for age and gender, were 292.5 [95% CI: (52.0–1645.5)] for the group “living alone” and were 89.4 [95% CI: (32.3–247.7)] for the group “living with family.” Although the model suggests that the influence of gender may be slightly opposite in the two groups, the predictive power of the LIMOS score is ultimately not significantly affected by age and gender (Table 5). However, in contrast to the group “living with family,” in the group “living alone” age still seemed to provide a certain independent explanatory value with regard to the discharge destination decision. Having selected the best model according to Akaike's information criterion, the resulting model suggested a possibly slight remaining impact for age in the group “living alone” although the LIMOS total score obviously appeared to be the clearly dominant factor. In the group “living with family” such a model selection procedure even suggested that the fact whether the LIMOS cut-off value had been exceeded or not could in itself sufficiently contribute the best explanation for the discharge destination decision.

**Table 4:** Logistic regression: discharge destination “home” predicted by LIMOS total discharge (> cut-off), adjusted for age and gender.

**Variable**	**Odds ratio**	**(95% CI)**	***p*-value**
**Group “living alone” (*****n*** **=** **137)**
LIMOS total discharge (>158 vs. ≤ 158)	292.5	(52.0–1645.5)	*p* < 0.001
Age (per year)	0.940	(0.878–1.005)	*p* = 0.071
Gender (male vs. female)	0.737	(0.148–3.671)	*p* = 0.71
**Group “living with family” (*****n*** **=** **418)**
LIMOS total discharge (>130 vs. ≤ 130)	89.4	(32.3–247.7)	*p* < 0.001
Age (per year)	0.980	(0.945–1.016)	*p* = 0.27
Gender (male vs. female)	1.664	(0.685–4.043)	*p* = 0.26

**Table 5:** Logistic regression: discharge destination “home” predicted by LIMOS total discharge (>cut-off), adjusted for age and gender.

**Variable**	**Odds ratio**	**(95% CI)**	***p*-value**
**Group “living alone” (*****n*** **=** **137)**
LIMOS total discharge (>158 vs. ≤ 158)	287.1	(52.0–1586.4)	*p* < 0.001
Age (per year)	0.944	(0.887–1.005)	*p* = 0.071
**Group “living with family” (*****n*** **=** **418)**
LIMOS total discharge (>130 vs. ≤ 130)	89.4	(32.8–243.9)	*p* < 0.001

*Proposed model [based upon model selection utilizing Akaike's information criterion (AIC)]*.

### Study 2—Verifying the LIMOS Cut-Off Scores

Based upon a second sample we verified the LIMOS cut-off scores for the groups “living alone” and “living with family” with regard to their sensitivity and specificity. This sample of 205 inpatients with stroke diagnosis was prospectively recruited between April 2018 until May 2020. They were admitted to the neurorehabilitation at the Neurocenter Luzerner Kantonsspital (LUKS) in Switzerland. The inclusion and exclusion criteria were the same as in the first study.

### Characteristics of the Second Patient Sample

One hundred and thirty-two patients with subacute stroke were included in this study (31.1% females), median age was 70 years old (Q1: 60, Q3: 76.75; range 22–89). Median time after stroke onset was 8 days (Q1: 6, Q3: 12.75; range 3–94 days). Ninety-nine (75%) patients had an ischemic and 33 (25%) a hemorrhagic stroke.

#### Overall Characteristics of the Two Groups “Living Alone” and “Living With Family”

**Figure 4:**
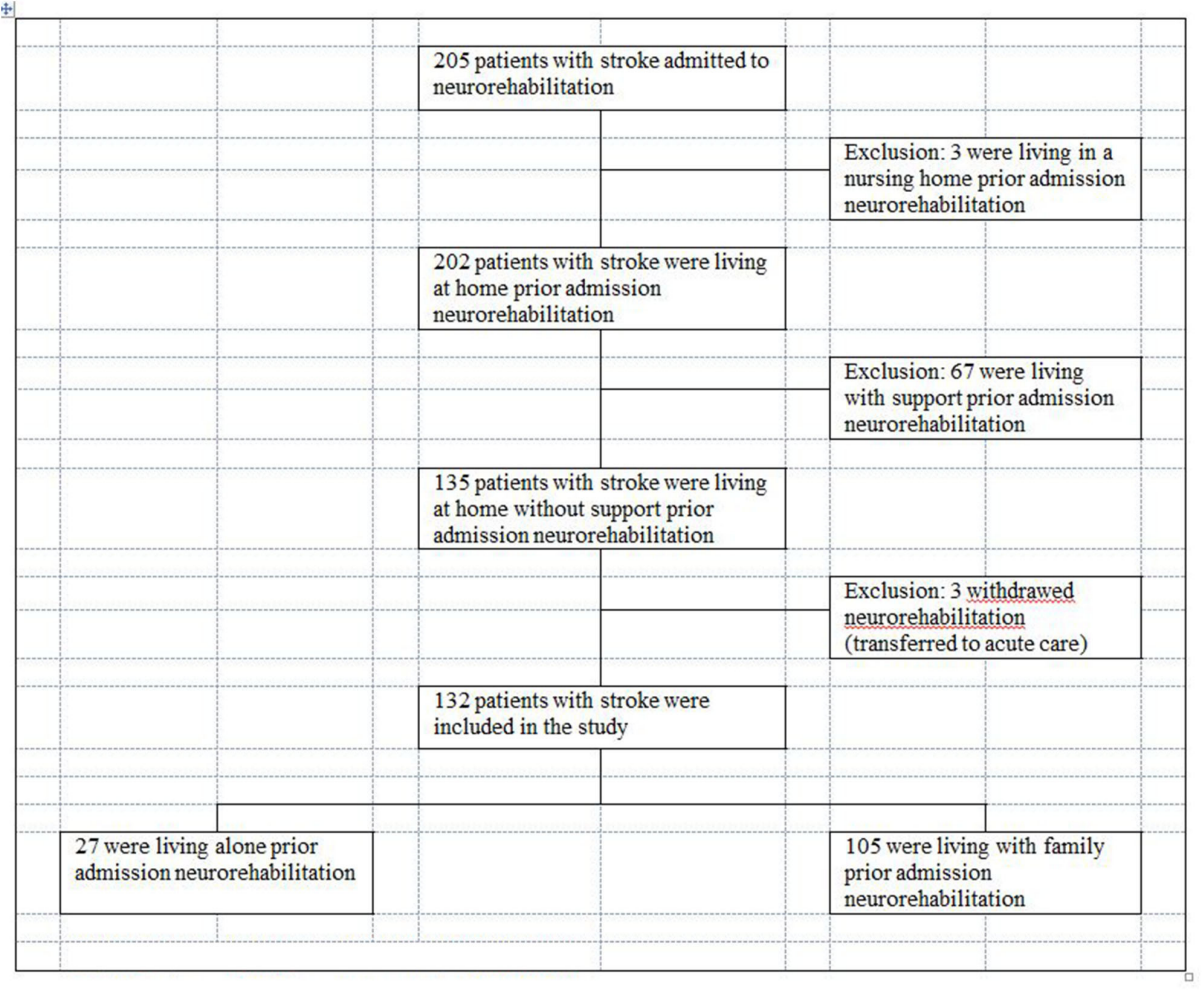
Flow chart—patient sample validation.

**Figure 5:**
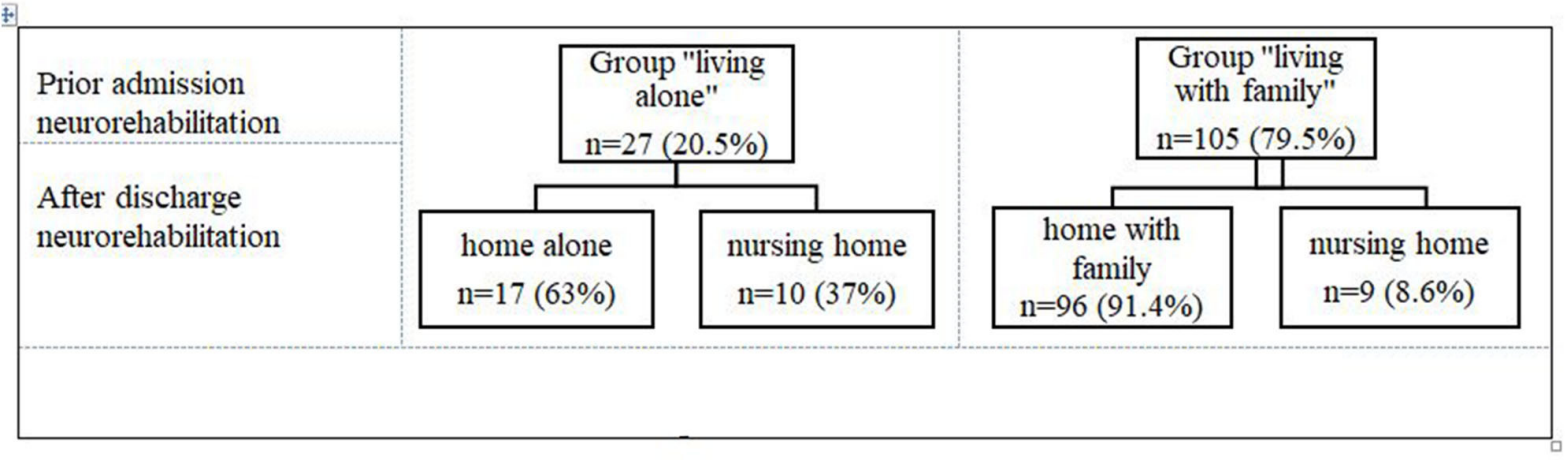
Living situation prior admission and after discharge from inpatient neurorehabilitation of the validation sample.

Prior to the admission for an inpatient neurorehabilitation, 27 (20.5%) of these patients were in the group “living alone” and 105 (79.5%) were in the group “living with family” (Figures 4, 5). At admission, both groups were comparable regarding their ADL impairments (median LIMOS total admission: “group living alone” 109 (Q1: 82.1, Q3: 159: range 46–218) vs. group “living with family” 138.33 (Q1: 103.71, Q3: 168.25; range 49–201) (*p* = 0.12). The group “living alone” was not significantly older (median age 72 years (Q1: 62, Q3: 79; range 31–89) than the group “living with family” (median age 69 years (Q1: 60, Q3: 76; range 22–89) (*p* = 0.46). However, significantly more women were in the group “living alone” 16 (59.3%) than in the group “living with family” 25 (23.8%) (*p* < 0.001).

For both groups, the chosen cut-off points resulted again in very high positive predictive values (100 and 98.9%, respectively). Likewise, in the first sample, the negative predictive value (66.7%) was lower in the group “living with family” (Table 6).

**Table 6:** Validation of best predictive LIMOS total discharge cut off scores (overall) for discharge destination home.

**Discharge destination “home”**	**Cut-off**	**Sensitivity**	**Specificity**	**PPV**	**NPV**
		**(95% CI)**	**(95% CI)**	**(95% CI)**	**(95% CI)**
**Overall (*****n*** **=** **132)**					
Group “living alone” (*n* = 27)	>158	0.941 (0.713–0.999)	1.000 (0.692–1.000)	1.000 (0.794–1.000)	0.909 (0.587–0.998)
Group “living with family” (*n* = 105)	>130	0.958 (0.897–0.989)	0.889 (0.518–0.997)	0.989 (0.942–1.000)	0.667 (0.349–0.901)

## Discussion

Using LIMOS as a sensitive scale (12), our study shows that stroke patients “living alone” had to achieve remarkably higher ADL cut-off scores at the end of neurorehabilitation than patients “living with family” in order to be discharged home after inpatient neurorehabilitation. Patients “living alone,” achieving a LIMOS cut-off score above 158 points were discharged home with a high degree of certainty. In contrast, for patients “living with family” a lower LIMOS cut-off score of 130 indicated diagnostic accuracy in predicting discharge home. In the second study, for both groups, the chosen cut-off points resulted again in very high positive predictive values (100 and 98.9%, respectively). Further analyses revealed that the predictive power of the LIMOS score was not significantly affected by age and gender.

The different LIMOS cut-off scores of both groups support the hypothesis that living alone is a high demand on stroke survivors and underlines the importance of giving special attention to them in the discharge planning (1, 3, 4, 8). Even if stroke survivors living alone receive some temporary support at home, whether from community or professional organizations or from family and friends who do not live in the same household, they critically lack a twenty-four-seven support. Selective support may not be sufficient to enable to live alone at home. Therefore, they need a certain degree of independency in performing activities themselves. The population of stroke survivors living alone is becoming increasingly relevant in everyday clinical practice, as the socio-demographic trend toward living alone is steadily increasing worldwide (10, 11, 18). Widowhood continues to be a large group of single households, but the number of younger people living alone is also increasing due to delayed or declining marriages, increasing divorces and increasing geographical mobility (11). On the other hand, depending on the cultural background, there is a possibility that people who previously lived alone are taken home into care by their relatives after a stroke.

In contrast to the group “living alone,” a high proportion of patients in the group “living with family,” were ultimately discharged home. Since the variables age and gender did not have a decisive impact, we assume that other factors play a role. One assumption is that family members can also be crucial in this process (4). This means that the willingness of family members to support patients at home must be given. In this study we did not analyse to what extent family members were able or willing to support the patients. If family members themselves already needed support, whether due to advanced age or illness, they may not be able to take the patients' home. On the other hand, family members may organize themselves in such a way that in some instances they can take home even a severely affected patient below a LIMOS 130-point limit and provide the necessary support.

The planning of discharge destination is a dynamic process during inpatient neurorehabilitation and depends on the patient's progress in performing ADL. The present study shows that observing ADL functions with LIMOS is sensitive for the prediction of discharge destination. Both LIMOS total cut-off scores help the clinicians to estimate whether the ADL performances are sufficient to be discharged home or not. Furthermore, they extend knowledge that an observation of ADL functions is sensitive for prediction of discharge destination (3, 5, 19).

The decision whether a patient can be discharged will always remain an individual judgement that has to be discussed between the patient, the patient's family (if applicable) and the responsible physician and the multidisciplinary team. In order to be able to put expectations into perspective—not least in situations that may be characterized by less realistic or overly optimistic hopes—a table can be helpful that indicates the probability that a certain LIMOS total score will, according to experience, lead to a discharge home. Supplementary File 1 provides such an overview: based on the 555 patients in the first sample, we estimated probabilities to reach discharge destination “home” by group (“living alone” or “living with family”). Enrichment with further data may possibly contribute to updates of this table.

Most of the previous studies used the FIM when evaluating ADL predicting discharge destination (1, 2, 4, 20–22). The FIM has some drawbacks that need to be considered, for instance ceiling and floor effects and the focus on physical domains or basic ADLs (13, 23). Another study investigated prediction of institutionalization of patients with stroke based on ICF with the WHODAS 2.0 assessment (24). WHODAS 2.0 is a self-reported instrument (24). The disadvantage of self–reported questionnaires is, however, that they strongly depend on preserved cognitive abilities (i.e., insight) of a stroke patient, possibly biasing the reliability of their answers.

A comprehensive ADL observation tool such as the LIMOS makes it possible to capture the patient in his entirety (12, 13). This demonstrates that stroke survivors in neurorehabilitation benefit from a support by a multidisciplinary team like neurologists, physiotherapists, occupational therapists, speech therapists, neuropsychologists, and nurses. In addition, our study also emphasizes the need of training not only at the level of functions, but above all of relearning and practicing ADLs if we want to enable stroke patients to return home.

A limitation of this study is that we do not know for how long the stroke survivors were able to live at home after discharge. A follow-up of the participants at a later time (for instance 6 months to 1 year later) would have been informative to verify how the stroke patients lived at home.

## Conclusion

To sum up, our study shows that people who live alone must perform ADL better than those who live with a family to be discharged home after stroke. For patients that live alone a LIMOS total score above 158 points is a strong indicator for a decision to be discharged home. On the other hand, for patients with a family a lower LIMOS score above 130 points can be sufficient. Neither age nor gender plays a significant role. In addition, our results enable monitoring the patient's process of improvement during a neurorehabilitation stay, and facilitate the decision concerning what discharge destination might be the most adequate.

## Data Availability Statement

The raw data supporting the conclusions of this article will be made available by the authors, without undue reservation.

## Ethics Statement

The studies involving human participants were reviewed and approved by Ethics committee of the State of Luzern (EKNZ/2017-00768), Switzerland. The patients/participants provided their written informed consent to participate in this study.

## Author Contributions

BO, DL, and TN contributed to conception, design of the study, and wrote sections of the manuscript. BO organized the database and wrote the first draft of the manuscript. BO and DL performed the statistical analysis. TP, TV, and TN critically revised for important intellectual content. All authors contributed to manuscript revision, read, and approved the submitted version.

## Conflict of Interest

The authors declare that the research was conducted in the absence of any commercial or financial relationships that could be construed as a potential conflict of interest.

## Abbreviations

ADL: Activities of daily living
AUC: Area under the ROC Curves
BI: Barthel Index
CI: Confidence Interval
ES: Effect Size
FIM: Functional Independence Measure
ICF, International Classification of Function: Health and Disability
LIMOS: Lucerne ICF-based Multidisciplinary Observation Scale
NPV: Negative predictive values
PPV: Positive predictive values
ROC: Receiver operating characteristics
SD: Standard Deviation
SRM: Standard response means.
