# Supplementary material for: Can I Discharge My Stroke Patient Home After Inpatient Neurorehabilitation? LIMOS Cut-Off Scores for Stroke Patients “Living Alone” and “Living With Family”
Source: Front Neurol. 2020 Nov 25;11:601725. doi: 10.3389/fneur.2020.601725 (PMC7732616; doi:10.3389/fneur.2020.601725)
Supplement: Supplementary file 1 [file Table_1.DOCX]

| **Supplementary Table 1 \|** Probability to reach discharge destination “home” by group ("living alone" or "living with family") *derived from a logit model explaining discharge destination by LIMOS Total at discharge* | | | |
| --- | --- | --- | --- |
| **LIMOS Total** | **«Living alone»** | **«Living with family»** |  |
| Sum Score | Cut off score 158 | Cut off score 130 |  |
| 225 | 99.9% | 100.0% |  |
| 220 | 99.8% | 99.9% |  |
| 215 | 99.7% | 99.9% |  |
| 210 | 99.5% | 99.9% |  |
| 205 | 99.3% | 99.8% |  |
| 200 | 98.9% | 99.8% |  |
| 195 | 98.3% | 99.7% |  |
| 190 | 97.3% | 99.6% |  |
| 185 | 95.9% | 99.4% |  |
| 180 | 93.7% | 99.2% |  |
| 175 | 90.5% | 98.9% |  |
| 170 | 85.9% | 98.5% |  |
| 165 | 79.6% | 97.9% |  |
| 160 | 71.4% | 97.2% |  |
| 155 | 61.5% | 96.1% |  |
| 150 | 50.6% | 94.8% |  |
| 145 | 39.6% | 92.9% |  |
| 140 | 29.5% | 90.6% |  |
| 135 | 21.1% | 87.5% |  |
| 130 | 14.7% | 83.5% |  |
| 125 | 9.9% | 78.7% |  |
| 120 | 6.6% | 72.9% |  |
| 115 | 4.3% | 66.1% |  |
| 110 | 2.8% | 58.7% |  |
| 105 | 1.8% | 50.8% |  |
| 100 | 1.2% | 42.9% |  |
| 95 | 0.8% | 35.4% |  |
| 90 | 0.5% | 28.5% |  |
| 85 | 0.3% | 22.5% |  |
| 80 | 0.2% | 17.4% |  |
| 75 | 0.1% | 13.3% |  |
| 70 | 0.1% | 10.0% |  |
| 65 | 0.1% | 7.5% |  |
| 60 | 0.0% | 5.6% |  |
| 55 | 0.0% | 4.1% |  |
| 50 | 0.0% | 3.0% |  |
| 45 | 0.0% | 2.2% |  |
