# Supplementary material for: Can I Discharge My Stroke Patient Home After Inpatient Neurorehabilitation? LIMOS Cut-Off Scores for Stroke Patients “Living Alone” and “Living With Family”
Source: Front Neurol. 2020 Nov 25;11:601725. doi: 10.3389/fneur.2020.601725 (PMC7732616; doi:10.3389/fneur.2020.601725)
Supplement: Supplementary file 2 [file Table_2.DOCX]

| **Supplementary Table 2 \|** The 45 ADL items of the Lucerne ICF-based Multidisciplinary Observation Scale (LIMOS) |
| --- |

| Factor 1  (interpersonal activity, self-care and motor) | 1. Basic interpersonal interactions (d710) |
| --- | --- |
|  | 1. Maintaining a body position (d415) 2. Changing basic body position (d410) 3. Transferring oneself (d420) 4. Lifting and carrying objects (d430) 5. Fine hand use (d440) 6. Hand and arm use (d445) 7. Walking (d450) 8. Walking long distances (d460) 9. Stairs (d455) 10. Moving around in another way (d455) 11. Using a wheelchair (d465) |
|  | 1. Washing oneself (d510) 2. Caring for body parts (d520) 3. Toileting (d530) 4. Dressing (d540) 5. Eating (d550) 6. Drinking (d560) 7. Looking after one’s health (d570 8. Coping night (d598) |
| Factor 2 (communication) | 1. Communication in general (d329) 2. Communication with – receiving – spoken messages (Understanding) (d315) 3. Communication with – receiving -written messages (Reading) (d325) 4. Speaking (d330) 5. Writing (d345) |
| Factor 3  (knowledge and general tasks) | 1. Acquiring basic skills (d1550) 2. Acquiring complex skills (d1551) 3. Focusing attention (d160) 4. Thinking (d163) 5. Solving simple problems (d1750) 6. Solving complex problems (d1751) 7. Remembering facts (d159) 8. Orientation (d179) 9. Visual spatial perception (d179) 10. Calculating (d172) 11. Making simple decisions (d177) 12. Making complex decisions (d177) |
|  | 1. Undertaking a simple task (d2100) 2. Undertaking a complex task (d2101) 3. Carrying out daily routine (d230) |
| Factor 4  (domestic life) | 1. Acquisition of goods and services (d620) 2. Preparing a simple meal (d6300) 3. Preparing a complex meal (d6301) 4. Doing household (d640) 5. Assisting others (d660) |

LIMOS Scores on a 5-point Likert Scale (minimum 45 points, maximum 225 points)

1 = patient is not able to fulfil a task or need assistance up to 75% (corresponding to “complete”)

2 = patient is able to fulfil tasks with assistance of 25% to 75% (corresponding to “severe”)

3 = patients is able to fulfil tasks with assistance less than 25% or under supervision (corresponding to

moderate”)

4 = patient is able to fulfil tasks independently but needs more time and/or with auxiliary materials, aids

corresponding to “slight”)

5 = patient is able to fulfil tasks independently (corresponding to “none”)
